# Pendelluft in Chronic Obstructive Lung Disease Measured with Lung Sounds

**DOI:** 10.1155/2012/139395

**Published:** 2012-03-21

**Authors:** Andrey Vyshedskiy, Raymond Murphy

**Affiliations:** Brigham and Women's, Faulkner Hospital, Boston, MA 02130, USA

## Abstract

*Objective*. The phenomenon of pendelluft was described over five decades ago. In patients with regional variations in resistance and elastance, gas moves at the beginning of inspiration out of some alveoli into others. Gas moves in the opposite direction at the end of inspiration. The objective of this study was to apply the method of lung sounds mapping, which is known to provide regional information about gas flow, to study pendelluft in COPD patients. *Methods*. A 16-channel lung sound analyzer was used to collect sounds from patients with COPD (*n* = 90) and age-matched normals (*n* = 90). Pendelluft at the beginning of inspiration is expected to result in vesicular sounds leading the tracheal sound by a few milliseconds. Pendelluft at the end of inspiration is expected to result in vesicular sounds lagging the tracheal sound. These lead and lag times were calculated for the 14 chest wall sites. *Results*. The lead time was significantly longer in COPD patients: 123 ± 107 ms versus 48 ± 59 ms in controls (*P* < 0.0001). The lag time was also significantly longer in COPD patients: 269 ± 249 ms in COPD patients versus 147 ± 124 ms in controls (*P* < 0.0001). When normalized by the duration of the inspiration at the trachea, the lead was 14 ± 13% for COPD versus 4 ± 5% for controls (*P* < 0.0001). The lag was 28 ± 25% for COPD versus 13 ± 12% for controls (*P* < 0.0001). Both lead and lag correlated moderately with the GOLD stage (correlation coefficient 0.43). *Conclusion*. Increased lead and lag times in COPD patients are consistent with the phenomenon of pendelluft as has been observed by other methods.

## 1. Introduction

There has been considerable interest in mechanical factors that affect the distribution of ventilation in patients with COPD. In the landmark study of Otis et al., it was shown that in normal young adults pulmonary compliance did not change over a wide range of breathing frequencies [1]. The authors inferred from this that the time constants are substantially the same for the separate pathways in normal lungs and that the distribution of ventilation is independent of breathing frequency. In contrast, pulmonary compliance dropped with increased breathing frequencies in normal subjects with induced bronchospasm and in patients with asthma and emphysema. The authors hypothesized that these observations were due to considerable time constants inequalities in separate pathways. Consequently, the authors believed that because of these factors the distribution of ventilation altered with changes of breathing frequency. Time constant inequalities of course are likely present in these patients during normal or deep breathing, and there is uneven distribution of ventilation in these instances. Indeed numerous studies using radioactive techniques have confirmed these observations [2]. Time constant inequalities cause transient movement of air between lung regions at the beginning of inspiration before air starts flowing at the trachea. At the end of inspiration, when gas flow at the trachea has just stopped, air moves in the opposite direction. This phenomenon is called pendelluft [1].

Multichannel lung sound mapping is known to provide regional gas flow information [3–5]. Since computerized lung sound analysis is noninvasive and can be readily used at the bedside its ability to document the magnitude of pendelluft can help in the diagnosis and monitoring of COPD. Pendelluft at the beginning of inspiration is expected to result in vesicular sounds leading the tracheal sound by a few milliseconds. Pendelluft at the end of inspiration is expected to result in vesicular sounds lagging the tracheal sound. In this paper, we examined the ability of computerized lung sound analysis to differentiate COPD patients from control subjects on the basis of these lead and lag measurements.

## 2. Materials and Methods

### 2.1. Patient Selection

Patients were selected for this study from a pool of patients who had undergone lung sound analysis as a part of a broader study of the correlation of disease processes with lung sounds patterns. The database includes more than one thousand patients on whom we have both the diagnosis and the lung sound analysis. To select patients for this study, we identified subjects with no recognized cardiopulmonary disorder as determined by the clinicians caring for these subjects (controls) and patients with chronic obstructive pulmonary disease (COPD) who were all outpatients and were seen by pulmonary specialists, Table 1.

**Figure 1:**
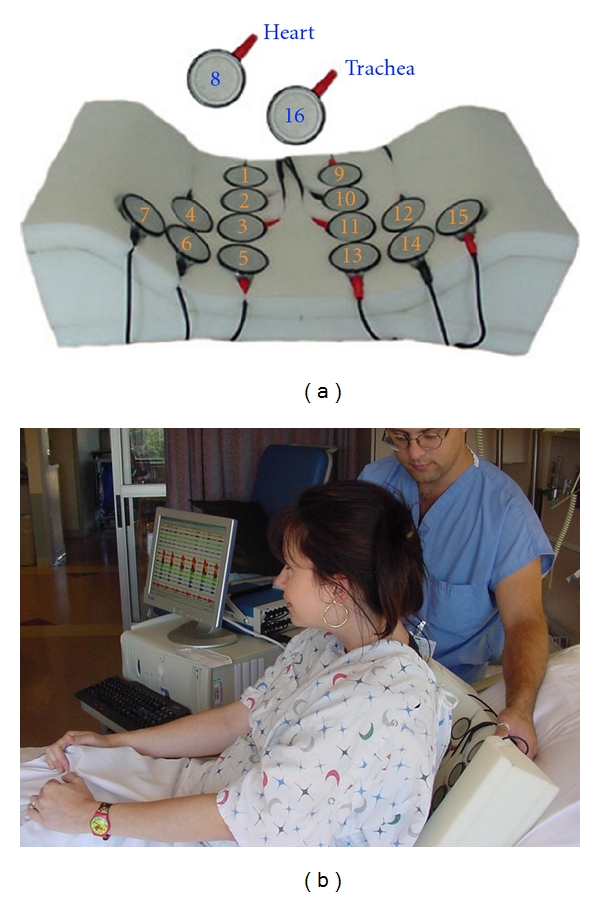
The multichannel lung sound analyzer: SteThoGraph or STG. (a) The arrangement of the microphones in the back pad arrayed over the posterior chest and lateral bases. Twelve microphones are placed on the back; numbers 1 through 6 are on the right side, 9 through 14 are on the left. There is one on each lateral base: microphone numbers 7 and 15, respectively. One microphone is over the trachea: number 16. (b) The placement of the soft foam pad containing the microphones.

Subjects were examined with a 16-channel lung sound analyzer (Stethographics Model STG1602), Figure 1. The STG has been described in detail in Murphy et al. and in Bergstresser et al. [6, 7]. In short, the STG uses electret microphones mounted in stethoscope chest pieces to record data on a PC. Fourteen microphones are incorporated into a soft foam microphone pad, Figure 1.

Ventilation was monitored with a tracheal microphone. This method provides an estimate of gas flow. At the flow rates we used in this study, tracheal gas flow is proportional to the root mean square of the sound amplitude: doubling the gas flow results approximately in doubling the root mean square of the sound amplitude [4, 8–14].

To avoid confusion between the inspiratory and expiratory phases, the start and end of the inspiratory phase was identified by the operator during recording based on the patient's abdominal movement.

Microphone 8 was used to record heart sounds, but these were not analyzed in this study. The microphone pad was positioned on a stretcher. Subjects were instructed to lie in a supine position on the microphone pad. Subjects were instructed to breathe more deeply than normal. Typically 3 to 6 full breaths were captured in a 20-second recording.

The results are presented as means ± SD unless otherwise stated. Student's *t*-test was used to compare the variables between the groups. Values of *P* < 0.05 were considered significant.

This study was approved by the institutional review board of Brigham and Women's/Faulkner Hospitals (protocol 2005-P-001559), and written consent was obtained from all patients or their next of kin.

### 2.2. Data Analysis

**Figure 2:**
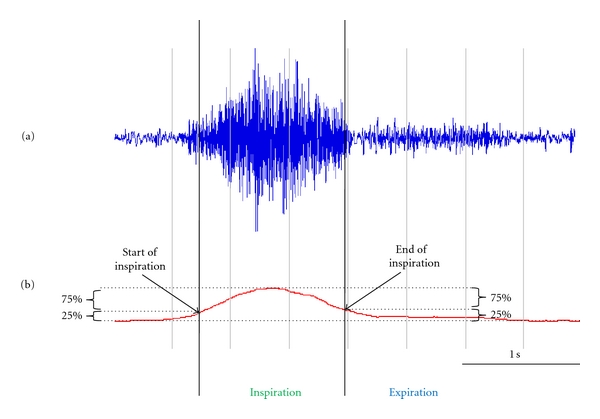
Identification of the start and end of inspiration. (a) A time-amplitude plot of a single channel breath sound band pass filtered between 80 Hz and 500 Hz. (b) The running average of the absolute value of the time amplitude signal. The start of inspiration was defined as the time when the signal reached 25% of its maximum level (left vertical line). The end of inspiration was defined as the time when the signal just dropped below 25% of its maximum value (right vertical line).

To identify the start and the end of inspiration on each channel, the lung sounds from the tracheal site and 14 chest channels were band pass filtered between 80 Hz and 500 Hz.  Figure 2(a) shows a time-amplitude plot of a single channel breath sound. The boxcar running average of the absolute value of the time amplitude signal was calculated and is presented in Figure 2(b). The width of the averaging window was 0.5 seconds. The start of inspiration was defined as the time when the signal reached 25% of its maximum level (Figure 2 left vertical line). The end of inspiration was defined as the time when the signal just dropped below 25% of its maximum value (Figure 2 right vertical line). The threshold of 25% was chosen arbitrarily.

**Figure 3:**
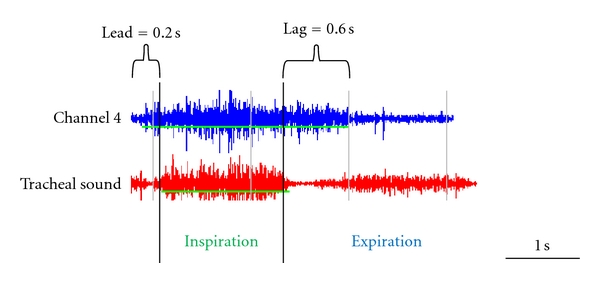
Calculations of the lead and lag. The thin horizontal green line under each channel waveform indicates the duration of inspiration at that channel. The thick vertical black lines indicate the beginning and the end of inspiration at the trachea.

The start and end of inspiration was identified in all 15 channels. The thin green line under each channel waveform indicates the duration of inspiration at that channel. The thick vertical black lines indicate the beginning and the end of inspiration at the trachea, Figure 3. The time of the start of the inspiration at the trachea was subtracted from the time of the start of the inspiration at each chest wall site to yield the lead time. In the example in Figure 3, the lead is 0.2 seconds. Similarly, the time of the end of the inspiration at the trachea was subtracted from the time of the end of the inspiration at each chest wall site to yield the lag time. In the example in the Figure 3, the lag is 0.6 seconds.

**Figure 4:**
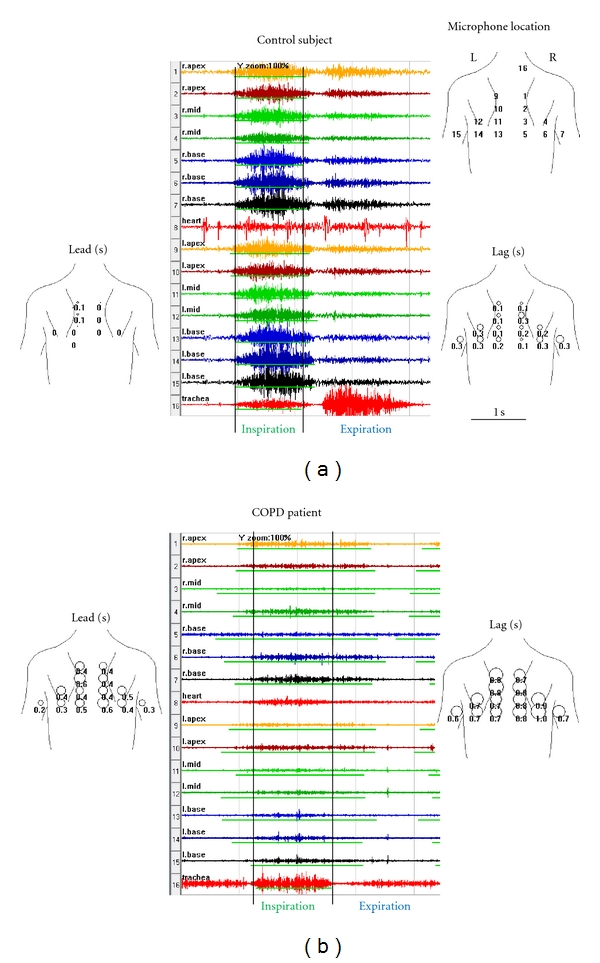
Comparison of sounds obtained from a control subject and a patient with COPD. Time amplitude plots of a single breath are displayed in stacked mode, center panel. The choice of colors is arbitrary and used here to aid in visual separation of channels. The thin green line under each channel waveform indicates the duration of inspiration at that channel as automatically identified by the STG software. Vertical lines mark the start and the end of inspiratory sound recorded at the trachea. Notice that in the control subject the inspiratory sound starts and ends at almost the same time at all the chest sites as well as the trachea. In COPD, inspiratory lung sounds at the chest sites lead the inspiratory sound at the trachea in the beginning of the inspiration. In addition, inspiratory lung sounds at the chest sites tend to lag the inspiratory sound at the trachea at the end of inspiration. The lead time at each chest wall site is displayed superimposed over the body plot, left panel. The circle diameter is proportional to the time delay. The lag time at each chest wall site is also displayed, right panel. The top right panel shows microphone location on the chest. In the control patient, the average lead was 15 ms or 1% and the average lag 199 ms or 17%. In the COPD patient, the average lead was 267 ms or 22% and the average lag 520 ms or 43%. In the control patient, the lead asynchrony was 2% and lag asynchrony was 18%. In the COPD patient, the lead asynchrony was 24% and lag asynchrony was 48%.

 The lead and lag averaged over all breaths are shown for each channel on the body plot, Figure 4. The circle diameter is proportional to the value of lead and lag measured at each channel.

To compare lead and lag between patients we averaged lead and lag over all chest channels. In addition, we also show lead and lag normalized by the duration of the inspiration at the trachea.

In addition to lead and lag calculations, we assessed asynchrony in 14 chest channels by calculating the standard deviation of lead and lag between the 14 chest channels. Note that asynchrony changes independent of lead and lag. Lung sounds leading the tracheal sound by a second in all chest channels result in a 1.0-second average lead and zero asynchrony. Lung sounds leading the tracheal sound by a second in just one chest channel result in 0.071 second average lead and 0.267 second asynchrony.

## 3. Results

Examples of sounds obtained from a control subject and a patient with COPD are shown in Figures 4(a) and 4(b), center panels. Vertical lines mark the start and the end of the inspiratory sound recorded at the trachea. Notice that in the control subject, Figure 4(a), the inspiratory sound starts and ends at almost the same time at all the chest sites as well as the trachea. In the COPD patient, Figure 4(b), the inspiratory lung sounds at the chest sites lead the tracheal sound in the beginning of inspiration and lag the tracheal sound at the end of inspiration. The lead and lag are quite variable between the channels.

The lead time is displayed superimposed over the body plot in Figure 4, left panels. The circle diameter is proportional to the time delay. The lag at each chest wall site is also displayed, Figure 4, right panels.

The means ± standard deviations of lead and lag averaged in all patients are presented in Table 2. Notice that both lead and lag are significantly greater in COPD than in controls (*P* < 0.0001).

The means ± standard deviations of lead and lag normalized by the duration of inspiration at the trachea are shown in Table 3.

The means ± standard deviations of asynchrony are shown in Table 4.

Lead, lag, and asynchrony demonstrated moderate correlation with GOLD stage and smoking history, Table 5. Lead, lag, and asynchrony demonstrated little correlation with patients age or gender, Table 5. 

## 4. Discussion 

In a normal subject, as the chest expands on inspiration, the lung uniformly dilates and the gas flow over the trachea is quickly initiated. In COPD, the lung dilatation is less uniform and air travels between lung regions due to regional variations in resistance and elastance before any significant gas flow is initiated at the trachea. This phenomenon, described over five decades ago by Otis et al., is called pendelluft [1]. 

Lung sounds are caused by gas flow in the lung and are affected by the pathophysiology of the tissues near where they are recorded. The amplitude of these sounds is generally believed to reflect ventilation of the lung. Indeed clinicians commonly use phrases like “good air entry” to describe auscultatory observations. There is considerable scientific support for this belief as studies of lung sounds have shown a correlation between acoustic measurements and flow rate [3–5, 8–13, 15, 16]. Although the clinical value of sound amplitude measurement has not been put into widespread use, one study showed that it was useful in guiding the amount of peep patients on ventilators should receive [5]. 

As it has been demonstrated that lung sounds can now be detected and quantified by computerized techniques, we set out to study pendelluft with lung sounds. Gas movement from some alveoli into others at the beginning of inspiration was assessed by calculating the difference in time between the start of the inspiratory sound at chest sensors and the start of inspiration over the trachea. Inspiratory sounds over the chest led the tracheal sound by 123 ± 107 ms in COPD patients compared to 48 ± 59 ms in controls (*P* < 0.0001). Similarly, at the end of inspiration sounds over the chest lagged the tracheal sound by 269 ± 249 in COPD patients compared to 147 ± 124 ms in controls (*P* < 0.0001). 

Both lead and lag correlated moderately with GOLD stage (correlation coefficient 0.43 for both) and smoking history (correlation coefficients 0.33 and 0.34). 

Asynchrony between the chest channels was also investigated. Asynchrony at the start of inspiration (lead asynchrony) was 16 ± 14% in COPD patients compared to 5 ± 6% in controls (*P* < 0.0001). Asynchrony at the end of expiration (lag asynchrony) was 32 ± 26% in COPD patients compared to 15 ± 13% in controls (*P* < 0.0001). Asynchrony correlated moderately with GOLD stage (correlation coefficient 0.43 for lead asynchrony and 0.46 for lag asynchrony) and smoking history (correlation coefficient 0.34 and 0.37). Physiologically asynchrony is likely to be related to pendelluft as it was highly correlated with lead and lag measurements (correlation coefficients 0.98 and 0.99, resp.). In other words, patients with high lead asynchrony also had high lead and patients with high lag asynchrony also had high lag. 

Otis and colleagues demonstrated that the pendelluft was facilitated by faster breathing [1]. Accordingly, we attempted to study the effect of panting on lead and lag. That effort was unsuccessful, however, as we were not able to reliably detect the start and end of inspiratory sounds. 

We do not believe that the increased lead and lag and increased asynchrony in COPD patients is due to muscle noise as the frequency of muscle noise is significantly lower than frequency of lung sounds: muscle noise power decays to near zero at about 60 Hz, while lung sounds reach maximum at about 100 to 150 Hz. Further we band pass filtered lung sounds between 80 Hz and 500 Hz. Therefore, muscle noise would have been filtered out before lead and lag analysis was performed. 

The finding that the degree of pendelluft can be characterized at the bedside using noninvasive technology that does not require radiation exposure or expensive equipment may aid researchers in performing a variety of studies that may help improve understanding of abnormal physiology. This includes study of regional inhomogeneity of ventilation and the factors associated with this common problem. Further studies could be done to investigate the relationship of lead and lag with ventilation heterogeneity as assessed by other techniques. In addition, intra- and interpatient variability should be studied as well as dependence of lead and lag on the respiratory rate and patient position. 

The diagnostic value of lead and lag was assessed by calculating their sensitivity and specificity in differentiating COPD patients from normal controls. Lead specificity was 0.96 and its sensitivity was 0.41. Lag specificity was 0.94 and its sensitivity was 0.42. Low sensitivity of lead and lag measurements makes them inefficient as sole measurements in the differential diagnosis of COPD. However, their high specificity makes lead and lag useful as additional markers of the disease. 

In summary, this technique, as it is simple to perform at the beside, has the potential in providing improved understanding of the pathophysiology of a variety of pulmonary disorders. 

## Figures and Tables

**Table 1:** The number of patients, their age and gender in each diagnostic category.

	Controls	COPD
Number of patients	90	90
Age (mean ± stdev)	69 ± 10	69 ± 11
Gender (% female)	50	50

**Table 2:** The means ± standard deviations of lead and lag.

	Controls	COPD	*P*
Lead (ms)	48 ± 59	123 ± 107	<0.0001
Lag (ms)	147 ± 124	269 ± 249	<0.0001

**Table 3:** The means ± standard deviations of lead and lag expressed as a percent of the duration of inspiration at the trachea.

	Controls	COPD	*P*
Lead (%)	4 ± 5	14 ± 13	<0.0001
Lag (%)	13 ± 12	28 ± 25	<0.0001

**Table 4:** The means ± standard deviations of asynchrony.

	Controls	COPD	*P*
Lead asynchrony (%)	5 ± 6	16 ± 14	<0.0001
Lag asynchrony (%)	15 ± 13	32 ± 26	<0.0001

**Table 5:** Correlation of lead and lag with clinical data.

	Lead	Lag	Lead asynchrony	Lag asynchrony
GOLD stage	0.43	0.43	0.43	0.46
Smoking index	0.33	0.34	0.34	0.37
Age	−0.08	0.04	−0.08	−0.02
Gender	0	0.16	−0.01	0.17
